# [Retracted] MicroRNA‑212 inhibits colorectal cancer cell viability and invasion by directly targeting PIK3R3

**DOI:** 10.3892/mmr.2023.13025

**Published:** 2023-05-30

**Authors:** Jian Zhang, Yongkang Zhang, Xiaoyun Li, Hongbo Wang, Quan Li, Xiaofeng Liao

Mol Med Rep 16: 7864–7872, 2017; DOI: 10.3892/mmr.2017.7552

Following the publication of this paper, it was drawn to the Editor's attention by a concerned reader that the western blotting data shown in Fig. 4A were strikingly similar to data appearing in different form in other articles by different authors at different research institutes, several of which have been retracted. Owing to the fact that the contentious data in the above article were already under consideration for publication prior to its submission to *Molecular Medicine Reports*, the Editor has decided that this paper should be retracted from the Journal. The authors were asked for an explanation to account for these concerns, but the Editorial Office did not receive a reply. The Editor apologizes to the readership for any inconvenience caused.

